# Volar approach combined with small dorsal incision in the treatment of AO type C distal radius fracture: A retrospective cohort study

**DOI:** 10.1097/MD.0000000000046624

**Published:** 2025-12-19

**Authors:** Xiang Yu, Wei-Hua Lu, Wei-wei Ma, Hai-Jian Lu, Rong-Guang Ao, Bing-Li Liu

**Affiliations:** a Department of Orthopedics, Shanghai Seventh People’s Hospital, Shanghai, China; b Department of Operating Theatre, Shanghai Seventh People’s Hospital, Shanghai, China.

**Keywords:** comminuted fracture, distal radius fracture, dorsal approach, plate internal fixation, volar approach

## Abstract

This study aims to explore the clinical efficacy of volar approach combined with small dorsal incision in the treatment of AO type C fracture of the distal radius. From January 2020 to December 2022, a total of 126 patients with AO type C fracture of the distal radius were treated. The treatment was conducted using a volar approach combined with a small dorsal incision. Initially, the Henry approach was employed from the volar side to expose the fracture site. Following reduction, Kirschner wires were used for temporary fixation. Subsequently, a small incision was made on the dorsal side to explore and assist in reducing the articular surface. Bone grafting was performed to support the collapsed articular surface, and Kirschner wires were again utilized to temporarily fix the joint surface bone fragments. Finally, a distal radius locking plate was positioned on the volar side. Routine rehabilitation exercises were initiated postoperatively, with follow-up visits scheduled at 1 month, 3 months, and 1 year after the procedure, accompanied by CT examinations. A total of 110 patients completed the follow-up, with an average duration of 13.8 months. All fractures achieved bony union at an average of 10.1 weeks. At the final follow-up, wrist function assessed by the Gartland and Werley scale was excellent in 68 cases, good in 33, and fair in 9. Radiographic parameters remained stable between postoperative and final follow-up assessments, with no significant differences in radial height (10.1 ± 3.6 mm vs 10.4 ± 3.5 mm, *P* > .05), radial inclination (20.8 ± 4.1° vs 20.4 ± 3.8°, *P* > .05), volar tilt (6.6 ± 4.4° vs 6.3 ± 4.6°, *P* > .05), or joint surface flatness (*P* > .05). Compared to the contralateral healthy side, the injured side exhibited significantly reduced mobility in both extension (70.8 ± 6.6° vs 82.5 ± 6.9°, *P* < .05) and flexion (70.6 ± 3.6° vs 87.5 ± 5.8°, *P* < .05). However, no significant differences were found in pronation (83.0 ± 4.7° vs 83.2 ± 4.1°, *P* > .05) or supination (85.5 ± 4.8° vs 85.7 ± 5.0°, *P* > .05). No major complications occurred. The volar approach combined with a small dorsal incision can achieve good clinical results in the treatment of AO type C fracture of the distal radius.

## 1. Introduction

Distal radius fractures are among the most prevalent clinical injuries, accounting for approximately one-sixth of all fractures in the human body.^[1,2]^ The incidence of these fractures has been increasing in recent years. Surgical intervention is often necessary for AO type C distal radius fractures that involve the radiocarpal articular surface.^[3]^ Currently, the most widely employed surgical technique in clinical practice is open reduction and internal fixation using locking plate via the volar approach.^[4]^ This method effectively exposes and stabilizes the bone fragments on the volar articular surface. Although the dorsal bone fragments can be successfully reduced by indirect reduction, some unstable fragments may experience postoperative displacement, leading to a poor prognosis.^[5,6]^ Some researchers have proposed creating auxiliary incisions on the dorsal side to facilitate direct visualization and reduction of the dorsal bone fragments, as well as the placement of dorsal auxiliary plate fixation to enhance the stability of these fragments.^[7]^ However, this approach carries a risk of extensor tendon irritation and may impose an increased financial burden on patients.^[8,9]^

In response to the aforementioned situation, we employ a volar approach with locking plate internal fixation, complemented by a small dorsal incision, to treat AO type C fracture of the distal radius. A minor incision is made on the dorsal side of the radius to facilitate exploration and assist in the reduction of the dorsal articular surface bone fragments. Bone grafting is conducted beneath the articular surface to support and stabilize these fragments. The volar plate and screws are utilized to reinforce the radiocarpal joint surface, with the screws being secured under direct visualization. This technique ensures that the screw length does not penetrate the dorsal cortex, thereby minimizing irritation to the dorsal tendons. Therefore, the purposes of this study were to introduce this modified technique and evaluate its clinical and radiographic outcomes.

## 2. Material and methods

### 2.1. Study subjects and design

This single-center, retrospective observational study analyzes 126 cases of AO type C fractures of the distal radius, all of which were admitted to the Orthopedics Department of our hospital between January 2020 and December 2022. Before enrollment, subjects were informed about the study protocol, Informed consent was obtained from all patients. All procedures in this study adhered to the ethical principles of clinical research outlined in the Declaration of Helsinki and received approval from the Medical Ethics Committee of Shanghai Seventh People’s Hospital (SSJW-2019110).

### 2.2. Inclusion and exclusion criteria

Inclusion criteria: age between 18 and 70 years, AO/OTA type C fractures of the distal radius, presence of a fresh closed fracture, and meeting the indications for open reduction and internal fixation (ORIF) recommended on the American Association of Orthopedic Surgeons (AAOS) standard.^[11]^ Exclusion criteria: presence of AO type A and type B distal radius fractures, concurrent fractures in other parts of the ipsilateral limb, associated neurovascular injury, involvement of significant organ damage, presence of ipsilateral elbow joint deformity due to congenital or acquired conditions, and pathological fractures.

### 2.3. Intervention measures

#### 2.3.1. Surgical method

**Figure 1.**
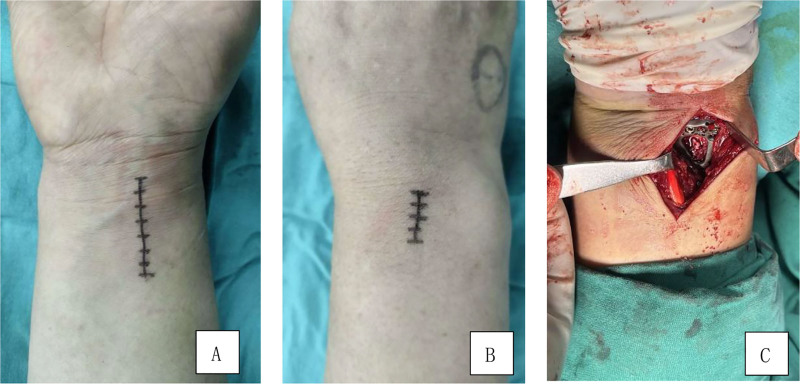
Surgical incision for distal radius fracture. (A) Volar approach, (B) dorsal approach, and (C) volar approach for plate placement.

Surgery was typically performed within 3 days of injury, and no later than 7 days. All procedures were conducted by senior surgeons and performed under general anesthesia. The affected limb was abducted and placed on a radiolucent side table, with a tourniquet applied to the upper arm. Using the modified volar Henry approach, an incision approximately 5 to 7 cm in length was made on the volar side of the wrist joint. The incision was made between the flexor carpi radialis muscle and the radial artery, with the flexor carpi radialis muscle retracted toward the ulnar side and the radial artery pulled laterally. The pronator quadratus muscle was then exposed, and a cut was made along the edge of the radius to reveal the fracture site. The radial diaphysis and volar articular surface bone fragments were reduced through pulling and prying, and were temporarily fixed using 2.0 mm Kirschner wire. Subsequently, a small incision approximately 2 to 4 cm in length was made on the dorsal side of the corresponding wrist joint, based on the position of the dorsal articular bone fragments as indicated by preoperative CT scan. The extensor retinaculum was cut, the fascial sheath was opened, and the dorsal bone fragments were exposed. After prying reduction, 1 to 3 mL particulate cancellous bone allograft (Shanghai Bio-Lu Biomaterials Co., Ltd., Shanghai, China) was implanted beneath the bone fragment and temporarily fixed with 1.2 mm Kirschner wires. An anatomical locking plate was then placed on the distal radius on the volar side, with screws of maximal length used to secure the articular surface bone fragments. Observation through the dorsal incision ensured that the screws do not penetrate the dorsal cortex. Reduction and fixation were verified using intraoperative C-arm imaging. Once fluoroscopy confirmed satisfactory results, the temporarily fixed K-wires were removed, and the stability of both the internal fixation and the distal radioulnar joint were assessed. The wound was irrigated with normal saline and closed layer by layer with sutures (Fig. 1).

#### 2.3.2. Postoperative treatment

Postoperative symptomatic treatment, including management of swelling and analgesia, was routinely administered, and the wrist joint was stabilized and protected with a brace. Finger joint flexion and extension exercises were initiated on the first day following surgery. The sutures were removed 2 weeks postoperation, at which point the wrist brace was discontinued, and wrist flexion and extension activities commenced.^[10]^ Based on the patient’s specific circumstances, the range of joint motion was gradually increased until normal daily activities were restored. The patient was advised to return to the hospital for follow-up evaluations at 1 month, 3 months, and 1 year after the operation, during which a CT scan of the wrist joint would be performed for all patients.

### 2.4. Outcome measures

#### 2.4.1. Gartland and Werley rating scale

The patient’s Gartland and Werley score^[11]^ at the last follow-up was recorded and divided into 4 grades: excellent, good, medium, and poor.

#### 2.4.2. Imaging evaluation

**Figure 2.**
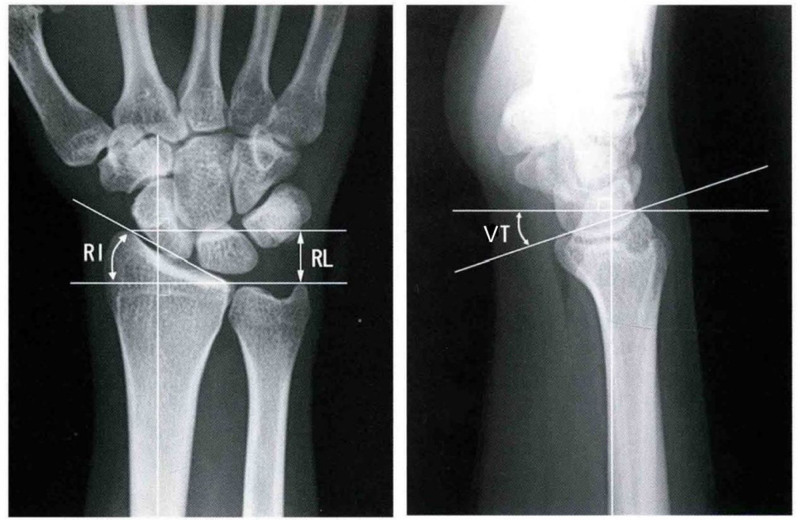
Measurements of radial height (RH), radial inclination (RI), and volar tilt (VT). RH = radial height, RI = radial inclination, VT = volar tilt.

The patients underwent X-rays and 3-dimensional reconstruction CT scans of the wrist joint 3 days post-surgery and again at the final follow-up.^[12]^ Measurements of radial height (RH), radial inclination (RI), volar tilt (VT), and joint surface flatness of the affected limb were recorded, and the differences in these parameters between the 2 time points were compared. The measurement methods for RH, RI, and VT are illustrated in Figure 2. The criteria for evaluating the flatness of the articular surface are as follows: an articular surface step of <1 mm and an articular surface bone gap of <1 mm are considered excellent; an articular surface step of 1 to 2 mm or an articular surface bone gap of 1 to 2 mm is classified as medium; and an articular surface step or gap between the facial bone fragments >2 mm is deemed poor.

#### 2.4.3. Wrist mobility

At the final follow-up, the range of motion of the wrist joint, including flexion, extension, pronation, and supination, was measured for both the injured and contralateral healthy sides using a universal standard goniometer with 1° increments. All measurements were performed by 2 experienced physical therapists who were blinded to the patient’s study details. Measurements of flexion and extension were taken in the sagittal plane. The axis of the goniometer was positioned near the radial styloid process of the wrist, with the stationary arm aligned with the long axis of the radius of the forearm, and the moving arm aligned with the long axis of the second metacarpal. Measurements of pronation and supination were conducted on a horizontal plane. The patient held a pencil, and a protractor was used to measure the angle between the pencil shaft and the vertical plane. Three consecutive measurements were taken for each direction of movement, and the average value was recorded and used for statistical analysis.

#### 2.4.4. Postoperative complications

Complications that arose in patients from the time of surgery to the final follow-up were documented. These complications included wound infection, screw loosening, screw penetration through the dorsal cortex, internal fixation breakage, fracture displacement, tendon rupture, and nerve injury.^[13,14]^

#### 2.4.5. Statistical analysis

Statistical analysis was performed using IBM SPSS Statistics, version 23.0 (Armonk). Data are presented as mean ± standard deviation. For quantitative data, analysis of variance or the Wilcoxon test was utilized, depending on the distribution of the data. The Cochran–Mantel–Haenszel (CMH) chi-square test was applied for ordered categorical data, while the Pearson chi-square test or Fisher’s exact probability method was employed for 2-category or unordered categorical data. A *P* value of <.05 was considered statistically significant.

## 3. Results

### 3.1. Baseline patient profiles

From January 2020 to December 2022, a total of 126 patients with AO type C fracture of the distal radius were treated. Of these, 110 patients completed the follow-up, while 16 patients did not adhere to follow-up protocols. Among the 110 patients, there were 18 males and 92 females. The ages of the patients ranged from 35 to 88 years, with an average age of 68.2 years. Bone mineral density T-scores varied from −3.6 to 1.2, yielding an average of −1.8. The causes of injury were categorized as follows: 15 cases resulted from traffic accidents, 9 cases were due to falls from a height, and 86 cases were attributed to falls on the ground. The mean interval from injury to surgery was 2.3 days (Table 1).

**Table 1:** Demographic data and clinical efficacy.

Number of people enrolled (cases)	110
Age (yr)	
Mean (min, max)	68.2 (35, 88)
Gender (cases)	
Male	18 (16.36%)
Female	92 (83.64%)
Bone density	
Mean (min, max)	−1.8 (−3.6, 1.2)
Causes of injury (cases)	
Traffic accidents	15
Falls from a height	9
Falls on the ground	86
Time from injury to surgery (d)	
Mean (min, max)	2.3 (0, 3)
Follow-up time (mo)	
Mean (min, max)	13.5 (12, 15)
Fracture healing time (wk)	
Mean (min, max)	12.1 (10, 20)
Gartland and Werley score (cases)	
Excellent (0–2)	68
Good (3–8)	33
Medium (9–20)	9
Poor (≥21)	0

### 3.2. Clinical efficacy

A total of 110 patients completed the follow-up, with the follow-up duration ranging from 12 to 15 months and an average follow-up time of 13.8 months. All patients achieved bony healing, with an average healing time of 12.1 weeks. At the final follow-up, wrist joint function was assessed using the Gartland and Werley scoring scale, which is categorized into 4 grades: scores of 0 to 2 were classified as excellent, with a total of 68 cases; scores of 3 to 8 were classified as good, with a total of 33 cases; scores of 9 to 20 were considered medium, with a total of 9 cases; and scores of ≥ 21 were deemed poor, with no cases recorded. Overall, 91.81% of patients (68 excellent + 33 good) demonstrated successful wrist function recovery (Table 1).

**Figure 3.**
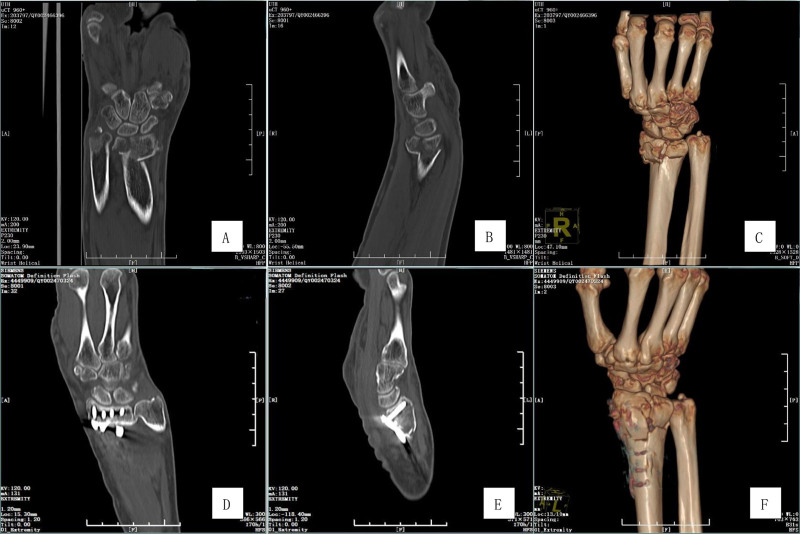
Imaging findings before surgery and 3 days after surgery. (A) Preoperative coronal CT revealed multiple fracture lines on the articular surface of the distal radius. (B) Preoperative sagittal CT indicated that the fracture of the distal radius articular surface is separated and exhibits steps. (C) Preoperative 3-dimensional CT demonstrated a fracture of the distal radius with dorsal comminution. (D) The coronal CT scan 3 days postoperation showed that the articular surface of the distal radius is flat, and the screws were evenly distributed. (E) The sagittal CT 3 days postoperation revealed that the articular surface of the distal radius had been reduced. The screws were positioned close to the articular surface, providing support, and their length extended just below the dorsal bone cortex. (F) The 3-dimensional CT 3 days postoperation indicated that the comminuted fracture on the dorsal side had been largely reduced. CT = computed tomography.

The patients underwent wrist CT scans 3 days post-surgery and at the final follow-up visit (Fig. 3). The CT scan performed 3 days after surgery revealed a RH of 10.1 ± 3.6 mm, a RI of 20.8 ± 4.1°, and a VT of 6.6 ± 4.4°. The assessment of joint surface flatness indicated excellent results in 87 cases, fair results in 23 cases, and no cases classified as poor. At the last follow-up, the RH measured 10.4 ± 3.5 mm, the RI was 20.4 ± 3.8°, and the VT was 6.3 ± 4.6°. The joint surface flatness was rated as excellent in 84 cases, fair in 26 cases, and again, no cases were classified as poor (Table 2). A comparison of RH, RI, VT, and joint surface integrity between the 2 CT scans showed no statistically significant differences (*P* > .05).

**Table 2:** Radiographic evaluation during follow-up (mean ± SD).

Variables	Postoperative		Final follow-up		*P* value
Radial height (mm)	10.1 ± 3.6		10.4 ± 3.5		.288
Radial inclination (°)	20.8 ± 4.1		20.4 ± 3.8		.144
Volar tilt (°)	6.6 ± 4.4		6.3 ± 4.6		.594
Joint surface flatness	Good	Medium	Good	Medium	.339
	87	23	84	26	

SD = standard deviation.

**Figure 4.**
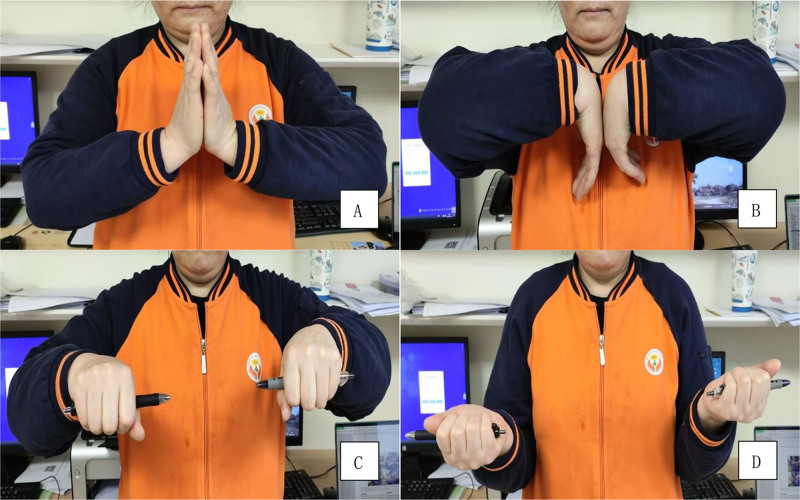
The range of motion in the right extremity of a 55-year-old female patient with an AO C type distal radius fracture demonstrated significant recovery. Extension (A), flexion (B), pronation (C), supination (D) of the wrist improved notably within 12 months following surgery.

In comparing the wrist joint mobility of the patient’s injured side with that of the contralateral side at the last follow-up (Fig. 4), the mean ranges of motion for the injured side were measured as follows: extension 70.8 ± 6.6°, flexion 70.6 ± 3.6°, pronation 83.0 ± 4.7°, and supination 85.5 ± 4.8° (Table 3). No significant differences were observed in pronation and supination when compared to the contralateral normal wrist (*P* > .05). However, statistically significant differences were noted in extension and flexion (*P* < .05).

**Table 3:** Comparison of the range of motion between the injured and the contralateral normal wrist at 12 months postoperatively (mean ± SD).

Variables	Injured side	Contralateral side	*P* value
Extension (°)	70.8 ± 6.6	82.5 ± 6.9	.022
Flexion (°)	70.6 ± 3.6	87.5 ± 5.8	.013
Pronation (°)	83.0 ± 4.7	83.2 ± 4.1	.660
Supination (°)	85.5 ± 4.8	85.7 ± 5.0	.443

SD = standard deviation.

During the follow-up period, no major complications, including incision infection, screw loosening, screw penetration through the dorsal cortex, internal fixation rupture, tendon rupture, or nerve injury, occurred in any patient. In 12 patients, a slight displacement of the dorsal bone fragment was observed during the follow-up CT scan conducted 1 month after surgery. This was managed conservatively with extended brace immobilization for 2 weeks. Upon reexamination, no further displacement was noted, and there was no significant functional impairment, indicating that this minor radiographic finding did not progress to a clinical complication.

## 4. Discussion

Distal radius fractures are among the most prevalent clinical injuries, accounting for approximately one-sixth of all fractures in the human body, and their incidence has been rising in recent years.^[15]^ For AO type C fractures involving the radiocarpal articular surface, surgical intervention is essential to achieve anatomical reduction and prevent long-term complications. Biomechanical studies emphasize that even minor articular step-offs >1 to 2 mm can lead to stress concentration, postoperative pain, and accelerated osteoarthritis.^[16,17]^ Any steps, separation, or displacement of the articular surface can lead to postoperative pain and restricted movement in the patient’s wrist joint, adversely affecting long-term prognosis.^[18]^ Traumatic wrist arthritis may arise from stress concentration on the articular surface.^[19]^ Biomechanical experiments have demonstrated that a step >2 mm on the radiocarpal joint surface can result in contact stress concentration.^[20]^

In contrast, our study demonstrated that the combined volar and minimal dorsal approach effectively achieved a flattened articular surface, with no cases exhibiting a step-off >2 mm and over 91% of cases rated as excellent or good in joint surface flatness at final follow-up (Table 2). Furthermore, while traditional dorsal augmentation techniques may risk extensor tendon irritation and increased costs, our method resulted in no such complications – specifically, no incidence of tendon rupture, nerve injury, or implant failure – highlighting its safety and efficacy in maintaining reduction without additional morbidity.^[21]^ Moreover, functional outcomes assessed using the Gartland and Werley scale showed that 91.81% of patients achieved excellent or good results, which is higher than the rates reported in studies utilizing volar locking plates alone for similar AO type C fractures.^[4]^ Postoperative range of motion measurements also indicated satisfactory recovery: mean extension of 70.8°, flexion of 70.6°, pronation of 83.0°, and supination of 85.5°. These values are within functional ranges and comparable to or better than outcomes achieved with more invasive dorsal plating techniques,^[6,22]^ further supporting the clinical superiority of our minimally invasive approach in restoring wrist function without increasing complication risks.

The surgical approaches for open reduction and locking plate internal fixation of distal radius fractures include volar, dorsal, and combined dorsal-volar approaches. Most scholars advocate the AAV (almost always volar) principle, which emphasizes the preference for accessing the palm side whenever possible.^[23,24]^ The volar approach is straightforward to execute and results in less soft tissue damage. The volar surface of the distal radius is flat, facilitating the placement of the steel plate. However, due to the anatomical characteristics of the radius, the dorsal bone cortex of the distal radius is significantly thinner than that of the volar side, leading to more severe crushing and displacement of dorsal fracture fragments compared to those on the volar side.^[15]^ Additionally, addressing the radial styloid process can be challenging with the volar approach, and it may not adequately manage cases involving displacement of the Lister tubercle fracture or compression of the tendon.^[25]^ For severely comminuted intra-articular fractures, reliance on the volar approach alone may hinder the reduction and fixation of all fracture fragments, thereby compromising surgical outcomes.^[26]^ With the introduction of the “three-column” theory of the wrist joint, many researchers have adopted the combined dorsal and palmar approach to effectively reduce and fix complex distal radius fractures, yielding favorable results. Mini-plate fixation on the dorsal side offers the advantage of secure fixation; however, it necessitates more extensive soft tissue dissection, which can lead to irritation of the dorsal extensor tendons, thereby increasing both surgical trauma and the economic burden on patients.^[7,27]^ In contrast, our technique of utilizing a small dorsal incision solely for assisted reduction and bone grafting – without placing additional dorsal hardware – avoided these pitfalls. Critically, none of the aforementioned complications, such as extensor tendon irritation, nerve injury, or implant-related issues, were observed in our cohort. This suggests that our minimally invasive approach achieves comparable stability while significantly reducing the risks associated with traditional dorsal augmentation.

Comminuted intra-articular fractures of the distal radius consist of 5 components: the radial styloid process, intra-articular incarceration, dorsal wall, volar edge, and dorsal ulnar fragment (DUF).^[28]^ In our cohort, DUF was identified in approximately 37.27% of cases (41/110) based on preoperative CT scan, thereby underscoring its significance in shaping our surgical strategy. Research indicates that the DUF is crucial for maintaining the stability of the distal radioulnar joint, preventing dorsal collapse of the distal radius, and ensuring appropriate palmar inclination.^[29,30]^ Currently, the prevailing consensus for the treatment of DUF in comminuted distal radius fractures advocates for the use of volar locking screw plate fixation. At least one screw is essential to prevent postoperative displacement.^[30]^ However, this surgical approach often results in irritation or rupture of the extensor tendon associated with the fractured fragment.^[9]^ To mitigate these complications, reducing the dorsal protrusion of the volar screw may be beneficial. Consequently, surgeons typically opt for distal locking screws that are slightly shorter than the measured length.^[31]^ Nonetheless, this adjustment to decrease the dorsal penetration of the screw may compromise the stability of the intra-articular fracture fragments, particularly the DUF, which can adversely affect the distal radius articular surface and sigmoid notch, leading to instability in the radiocarpal and distal radioulnar joints.^[32]^

In summary, our technique of utilizing a small dorsal incision for assisted reduction and bone grafting – without dorsal hardware – achieved direct visualization for anatomical articular reconstruction, secure fixation through volar screw length monitoring, and minimized extensor tendon irritation. Postoperative CT scans confirmed adequate stability of the articular fragments, with only 10.91% of cases (12/110) exhibiting minor asymptomatic displacement that did not affect functional outcomes, ultimately yielding a 91.81% rate of excellent/good wrist function. Despite these promising results, several limitations should be acknowledged: This study was conducted at a single institution, potentially limiting the generalizability of the findings. The retrospective design may introduce selection bias and unmeasured confounding factors. While adequate for preliminary analysis, a larger sample size would strengthen the statistical power and allow for more robust subgroup analyses. The mean follow-up of 13.8 months may be insufficient to evaluate long-term complications such as post-traumatic osteoarthritis or late implant failure. Our study cohort exhibited a demographic skew, with a high proportion of female patients (85.5%) and an average bone mineral density T-score of −1.8, indicating a population predominantly characterized by osteopenia. While this reflects the typical epidemiology of fragility fractures of the distal radius in the elderly population, it may limit the generalizability of our findings to younger, male patients, or those with normal bone quality. Future multi-center, prospective randomized controlled trials with longer follow-up periods are warranted to validate these findings and further establish the efficacy of this technique.

## 9. Conclusion

This study demonstrates that the volar approach combined with a small dorsal incision represents an effective surgical strategy for managing AO type C distal radius fractures. The technique facilitates anatomical reduction of articular fragments through direct visualization while minimizing soft tissue disruption. Postoperative outcomes confirmed excellent functional recovery with maintained radiographic alignment and an absence of implant-related complications. Our findings support the clinical efficacy of this combined approach, which offers a viable alternative for achieving stable fixation and optimal functional outcomes in complex intra-articular fractures of the distal radius.

## Author contributions

**Investigation:** Xiang Yu, Wei-Hua Lu.

**Methodology:** Xiang Yu, Wei-Hua Lu, Wei-wei Ma, Hai-Jian Lu, Rong-Guang Ao, Bing-Li Liu.

**Resources:** Wei-wei Ma, Hai-Jian Lu, Bing-Li Liu.

**Supervision:** Xiang Yu, Bing-Li Liu.

**Project administration:** Rong-Guang Ao, Bing-Li Liu.

**Writing – original draft:** Xiang Yu, Wei-Hua Lu.

**Writing – review & editing:** Rong-Guang Ao, Bing-Li Liu.
